# Role of Reactive Oxygen Species in the Neural and Hormonal Regulation of the PNMT Gene in PC12 Cells

**DOI:** 10.1155/2011/756938

**Published:** 2011-10-09

**Authors:** James A. G. Crispo, Dominique R. Ansell, Gino Ubriaco, T. C. Tai

**Affiliations:** ^1^Medical Sciences Division, Northern Ontario School of Medicine, Sudbury, ON, P3E 2C6, Canada; ^2^Department of Chemistry & Biochemistry, Laurentian University, Sudbury, ON, P3E 2C6, Canada; ^3^^2^ Department of Biology, Laurentian University, Sudbury, ON, P3E 2C6, Canada; ^4^Biomolecular Sciences Programme, Laurentian University, Sudbury, P3E 2C6, Canada

## Abstract

The stress hormone, epinephrine, is produced predominantly by adrenal chromaffin cells and its biosynthesis is regulated by the enzyme phenylethanolamine N-methyltransferase (PNMT). Studies have demonstrated that PNMT may be regulated hormonally via the hypothalamic-pituitary-adrenal axis and neurally via the stimulation of the splanchnic nerve. Additionally, hypoxia has been shown to play a key role in the regulation of PNMT. The purpose of this study was to examine the impact of reactive oxygen species (ROS) produced by the hypoxia mimetic agent CoCl_2_, on the hormonal and neural stimulation of PNMT in an in vitro cell culture model, utilizing the rat pheochromocytoma (PC12) cell line. RT-PCR analyses show inductions of the PNMT intron-retaining and intronless mRNA splice variants by CoCl_2_ (3.0- and 1.76-fold, respectively). Transient transfection assays of cells treated simultaneously with CoCl_2_ and the synthetic glucocorticoid, dexamethasone, show increased promoter activity (18.5-fold), while mRNA levels of both splice variants do not demonstrate synergistic effects. Similar results were observed when investigating the effects of CoCl_2_-induced ROS on the neural stimulation of PNMT via forskolin. Our findings demonstrate that CoCl_2_-induced ROS have synergistic effects on hormonal and neural activation of the PNMT promoter.

## 1. Introduction

Epinephrine is synthesized by the catecholamine biosynthetic enzyme phenylethanolamine N-methyltransferase (PNMT, EC 2.1.1.28) [1]. PNMT is a critical determinant of epinephrine production in adrenal chromaffin cells during acute and chronic stress. Stress contributes to the pathophysiology of many diseases and epinephrine and glucocorticoids (cortisol and corticosterone) are the major stress hormones that initiate the biological responses permitting the organism to cope with adverse physiological, psychological, and environmental stress [2]. All catecholamine biosynthetic enzymes including PNMT are stress responsive; however, their responses are stressor-specific, dependent on stress intensity, duration, and number of repeated exposures [3, 4]. 

The PNMT gene has been shown to be both hormonally and neurally regulated through activation of the hypothalamic-pituitary-adrenal (HPA) axis and the sympathoadrenal (SA) system [5]. Both activation mechanisms exert transcriptional and post-transcriptional influences on the PNMT gene [6–8]. Hormonal activation of the PNMT gene is dependent on extremely high concentrations of glucocorticoids which induce transcriptional changes via glucocorticoid response elements (GREs) upstream of PNMT transcription initiation site [9]. The activation of PNMT through the sympatho-adrenal system can occur via the release of acetylcholine and PACAP from the splanchnic nerve [5]. Neurotransmitters such as acetylcholine, serotonin, and the peptide neurotransmitter PACAP have been shown to induce PNMT via the protein kinase A (PKA) and protein kinase C (PKC) pathways [10, 11]. In addition, both acetylcholine and PACAP activate signaling cascades that regulate transcription factors expressed exclusively in adrenergic cells such as the early growth response transcription factor 1 (Egr-1) and promote PNMT transcription [12]. 

Previous studies have shown that hypoxia is a potent stressor involved in the regulation of PNMT [13]. Cells experiencing lowered O_2_ levels (hypoxia) undergo a variety of biological responses in order to adapt to these unfavorable conditions [14]. Hypoxia, or decreased oxygen concentration, activates a variety of complex pathways at both the cellular and organism level with the ultimate aim of reinstating oxygen homeostasis. Although physiological responses to hypoxia have been appreciated for a long time, the molecular processes activated within the cells are still under investigation. However, this area has been greatly advanced by the discovery of a class of transcription factors that respond to hypoxia, HIF (hypoxia inducible factors) [15]. The HIFs stimulate a variety of genes, including PNMT [16–19]. 

Hypoxic conditions can also give rise to the production of reactive oxygen species (ROS) as well as reactive nitrogen species (RNS). The production of ROS/RNS is referred to as oxidative stress, a condition in which the balance between production and disposal of ROS/RNS is altered [20]. Several studies report that exposing cells or tissues to hypoxia increases oxidative stress and that this increase is generated by the mitochondria [21]. The ROS and RNS that arise from hypoxic conditions can lead to fixed changes in signal transduction and gene expression, resulting in disease development and progression [22]. ROS/RNS function as specific signaling molecules to trigger the activation of specific transduction pathways and damage to cellular components. ROS/RNS mediate these effects through the activation of specific transcription factors to control the transcription of a range of target genes. Several studies demonstrate that the delivery of CoCl_2_ to cultured cells can mimic hypoxic responses, including the increased production of ROS [22, 23]. Additionally, exposure of PC12 cells to hypoxia-mimicking concentrations of CoCl_2_ has been shown to upregulate the transcription of HIF1*α* and cause mitochondrial DNA damage [24]. 

The purpose of this study is to understand the role of oxidative stress arising from a hypoxic environment, and its effect on the hormonal and neural stimulation of PNMT. The effects of oxidative stress on PNMT and thereby epinephrine have not previously been shown and will allow for a better understanding of the impact of oxidative stress on the transcriptional machinery involved in the regulation of the PNMT gene. This, in turn, will allow us to better understand how epinephrine production is controlled via PNMT and its role as a neuroendocrine regulator in various disease states. The present study shows that under mimetic hypoxic stress generated via CoCl_2_, PC12 cells elicit the production of ROS and promote PNMT gene transcription. Additionally, oxidative stress arising from the hypoxia mimetic agent CoCl_2_ further promotes the transcription of the PNMT gene when combined with hormonal as well as with neural stimulation.

## 2. Materials and Methods

### 2.1. Cell Culture

PC12 cells (from D. O'Connor, UCSD, San Diego, CA, USA) were cultured in DMEM supplemented with 5% equine serum, 5% bovine serum and gentamycin sulphate (50 *μ*g/mL). All cells were maintained in a humidified incubator at 37°C in an atmosphere of 5% CO_2_–95% air and grown to 80–90% confluency before being passed or used in an experiment. Hypoxia was achieved by displacement of oxygen from 21% to 5% with nitrogen gas. Prior to experimentation, cells were transferred to DMEM containing charcoal-treated serum. For transfection studies, cells were plated in 24-well tissue culture plates at a density of 4-5 × 10^5^ cells/well. For total RNA extraction, cells were grown in 100-mm culture dishes to a density of 5 × 10^5^–1.8 × 10^6^ cells per dish. Following seeding, cells were allowed to adhere to plates for 16–24 h prior to beginning experiments. Cells were then drug treated with CoCl_2_ (200 *μ*M), dexamethasone (1 *μ*M), N-acetyl-L-cysteine (5 mM) (Sigma-Aldrich, St. Louis, MO, USA), forskolin (10 *μ*M) (LC Laboratories, Woburn, MA, USA) or the combination of each agonist with CoCl_2_ for times and at concentrations specified in the figure legends.

### 2.2. Measurement of Intracellular ROS by Flow Cytometry

Production of intracellular ROS was quantified using the CM-H_2_DCFDA method (Invitrogen, C6827). Following treatments, culture media was collected in 15 mL conical tubes. Cells were then scraped with 1 mL cold phosphate-buffered saline (PBS, pH 7.4) and added to the conical tubes, centrifuged at 1,000 rpm (5 min, 4°C) and washed with cold PBS (pH 7.4). Cell pellets were resuspended in 1 mL 2.5 *μ*M detection reagent, transferred to 1.5 mL centrifuge tubes and incubated at 37°C for 30 mins. Cells were then pelleted, resuspended in 1 mL culture medium, and incubated at 37°C for 30 min. Following incubation, cells were centrifuged at 2,400 rpm (8 min, 4°C) and washed with PBS (pH 7.4). Cells were then resuspended in 1 mL PBS (pH 7.4) and analyzed using a flow cytometer (BD Biosciences, FACS Canto II). In brief, cells were excited with 488/10 nm, and their fluorescence emission was recorded in the 530/30 nm range. The amount of intracellular ROS within cell populations is directly proportional to their mean fluorescent emission. Fluorescent emission values from 10,000 independent events were used to compute a mean fluorescent value for each treatment group. Treatment groups were then compared to stained control cells to determine the relative fold change of intracellular ROS.

### 2.3. Measurement of Nitric Oxide

Extracellular nitric oxide levels were quantified using the Nitric Oxide Analyzer (model 280, Sievers Instruments, Boulder, Colorado) [25]. Following treatments, culture media was collected in 1.5 mL centrifuge tubes. In brief, 10 *μ*L of media from each treatment was injected into a high-sensitivity detector. This device uses the chemiluminescent method to determine media content of nitrite (NO_2_^●^) and nitrate (NO_3_^●^), the stable oxidation products of NO. The nitroso-compounds NO_2_^●^ and NO_3_^●^ are reduced to NO by exposure to vanadium chloride which is then determined from the gas-phase chemiluminescent reaction between NO and ozone. Emission from electronically excited nitrogen dioxide is in the red and near red infrared region of the spectrum and is detected by a thermoelectrically cooled, red-sensitive photomultiplier tube. A calibration curve using sodium nitrate standards was used to calculate concentrations for the samples.

### 2.4. RT-PCR

PC12 cells (1.6 × 10^6^ cells/mL) were seeded into 60-mm culture dishes in modified DMEM, and treated as previously described. Cells were harvested and lysed in 500 *μ*L Tri-Reagent (Sigma Aldrich Canada Ltd) and total RNA isolated per manufacturer's protocol. Total RNA pellets were resuspended in diethylpyrocarbonate-treated nuclease-free water and concentrations determined using spectrophotometric measurements of absorbance at 260 nm (NanoDrop; Nanodrop Technologies, Wilmington, DE). 

2 *μ*g of total RNA was treated with DNase I (Sigma Aldrich Ltd.) following manufacturer's protocol, and cDNA subsequently synthesized using 100 U Revert Aid Moloney Murine Leukemia Virus reverse transcriptase enzyme as per manufacturer's protocol (Fermentas, Burlington, ON, Canada). PCR was performed in 25 *μ*L reaction volumes containing 78 ng of cDNA, using 50 U GoTaq Flexi DNA polymerase (Promega) containing 200 *μ*M of dNTPs, 1.5 mM MgCl_2_, and 25 ng of forward and reverse primer sequences specific for the following genes: PNMT and glyceraldehyde 3-phosphate dehydrogenase (GAPDH) (Table 1). 

PCR products (10 *μ*L) were electrophoresed on a 1.5% agarose gel in 40 mM Tris-acetate and 2 mM EDTA buffer (pH 8), stained with ethidium bromide, documented using the Chemidoc XRS (Biorad) imaging system and densitometric analysis performed using the Quantity One software (Biorad). 

### 2.5. Plasmids

The wild-type PNMT promoter-luciferase reporter gene construct pGL3RP893 was generated as previously described [7, 26, 27]. Competent E. coli cells were transformed with plasmid DNA to generate the PNMT promoter-luciferase reporter gene construct, and DNA plasmids were subsequently purified using the Invitrogen PureLink HiPure Plasmid DNA purification kit (Invitrogen Invitrogen Life Technologies Corp. Burlington, ON, Canada).

### 2.6. Transient Transfection Assays

Transient transfections were performed using the polyethylenimine (PEI) method as previously described [7, 28]. PC12 cells grown in 24-well tissue culture plates were transfected with 1.0 *μ*g of wild-type promoter-luciferase reporter gene construct using 5 *μ*L of 1X polyethylenimine to transfect 1 *μ*g of plasmid with an incubation period of 3 h. After transfection, the cells were washed with phosphate buffered saline (PBS, pH 7.4) and maintained in culture medium for 24 h, followed by drug treatment.

### 2.7. Luciferase Assays

Cell culture medium was removed, the cells rinsed twice with PBS and then lysed with 100 *μ*L lysis buffer (1.25 mM Tris-phosphate, pH 7.8, 2 mM DTT, 2 mM 1,2-diaminocyclohexane-N,N,N′,N′-tetraacetic acid 10% Glycerol and 1% Triton X-100) and subjection to a freeze-thaw cycle. Cell lysates were centrifuged at 3000 g for 10 min, and 20 *μ*L of supernatant assayed for luciferase activity as previously described by Tai and Wong using a microplate luminometer (Fluostar Optima BMG Labtech Nepean, ON, Canada) [7]. Total protein in the lysates was determined by the method of Bradford (1976), and luciferase activity expressed as optical density units per *μ*g of protein [29].

### 2.8. Data Analysis

All data are presented as the mean ± SEM. Experiments were repeated at least three times and statistical significance between experimental and control groups was determined by one-way ANOVA followed by a posthoc comparison using the Student-Newman-Keuls for multiple comparisons test. Results were considered statistically significant with a *P* < 0.05.

## 3. Results

### 3.1. Effect of CoCl_2_ and Hypoxia on Intracellular ROS and NO Levels

**Figure 1:**
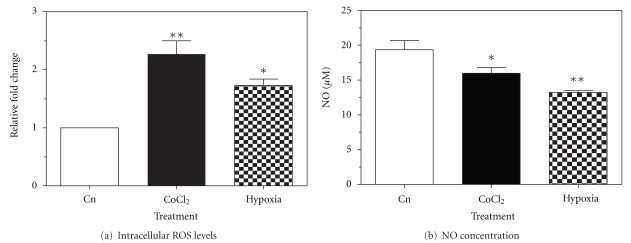
Effect of CoCl_2_  and hypoxia exposure on free radicals. Levels of intracellular ROS (a) and extracellular NO (b) were measured in PC12 cells following 24 h exposure to either 200 *μ*M CoCl_2_ or 5% oxygen at 37°C. Data presented as relative fold change or NO concentration ± SEM from a minimum of three independent experiments. Significance defined as *P < 0.05; **P < 0.01.

PC12 cells treated with 200 *μ*M CoCl_2_ or exposed to 5% oxygen for 24 h increased intracellular ROS levels 2.3-fold (*P* < 0.01) and 1.7-fold (*P* < 0.05), respectively, compared to control (Figure 1(a)). Conversely, PC12 cells treated with CoCl_2_  or exposed to 5% oxygen for 24 h showed a significant decrease of 3 *μ*M (*P* < 0.05) and 6 *μ*M (*P* < 0.01) in intracellular NO levels, respectively, compared to control (Figure 1(b)).

### 3.2. Effect of NAC on CoCl_2_  Induced Gene Expression and on Intracellular ROS and NO Levels

**Figure 2:**
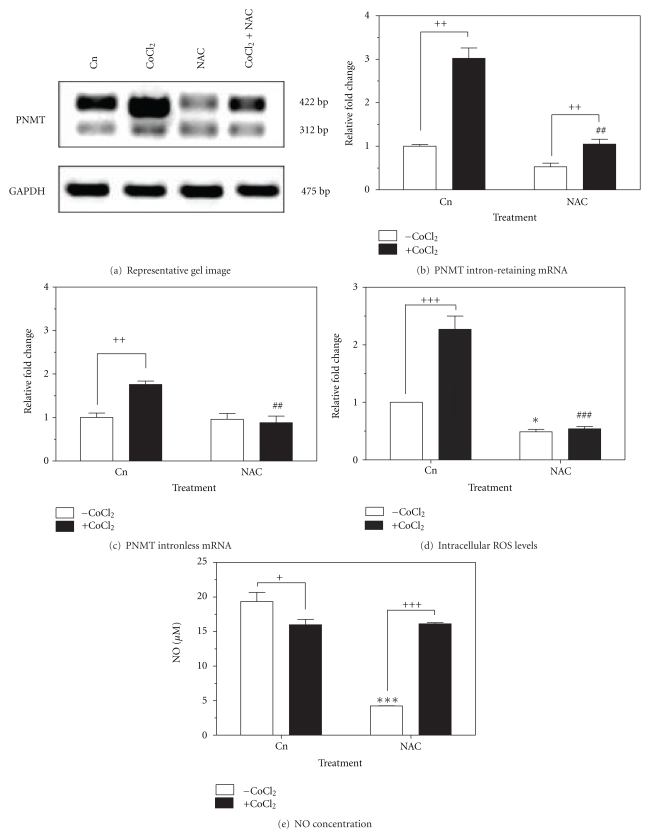
Effect of NAC pretreatment on levels of PNMT mRNA, intracellular ROS, and extracellular NO in PC12 cells treated with 200 *μ*M CoCl_2_ for 24 h at 37°C. (a) Representative image (*n* = 3) of PNMT (intron-retaining and intronless splice variants) mRNA levels from semiquantitative RT-PCR. GAPDH mRNA was used as a housekeeping control. (b) PNMT intron-retaining mRNA and (c) intronless mRNA. Data is normalized to GAPDH control. (d) Intracellular ROS levels and (e) NO concentration after 24 h. Data presented as relative fold change or NO concentration ± SEM from a minimum of three independent experiments. Significance defined as ^∗/+^ *P* < 0.05; ^++/##^ *P* < 0.01; ^∗∗∗/+++/###^ *P* < 0.001.

CoCl_2_ treatment significantly increased PNMT intron-retaining and intronless mRNA levels by 3.0-fold (*P* < 0.01) and 1.76 (*P* < 0.01) respectively, compared to untreated control. To assess whether mRNA induction of PNMT by CoCl_2_ could be abolished by antioxidant treatment, cells were pretreated with 5 mM NAC for 30 min prior to CoCl_2_ delivery (Figures 2(a), 2(b), and 2(c)). Cells pretreated with NAC prior to CoCl_2_ delivery showed decreased PNMT intron-retaining and intronless mRNA levels by 2.0-fold (*P* < 0.01) and 0.9-fold (*P* < 0.01), respectively, compared to CoCl_2_ controls (Figures 2(b) and 2(c)).

To confirm the scavenging of ROS by NAC during CoCl_2_  stress, intracellular ROS levels were measured after 24 h. NAC treatment significantly reduced basal levels of intracellular ROS by 0.5-fold (*P* < 0.05) and significantly reduced levels of CoCl_2_-generated ROS by 1.8-fold (*P* < 0.001) compared to control (Figure 2(d)). Cells treated with 5 mM NAC reduced basal NO levels by 15 *μ*M (*P* < 0.001), while cells treated with CoCl_2_ alone significantly reduced NO levels by 3.4 *μ*M compared to control. Cells pretreated with NAC prior to CoCl_2_ treatment showed no significant change in NO levels compared to cells treated with CoCl_2_ alone (Figure 2(e)).

### 3.3. Effect of ROS on the Hormonal Regulation of the PNMT Gene

**Figure 3:**
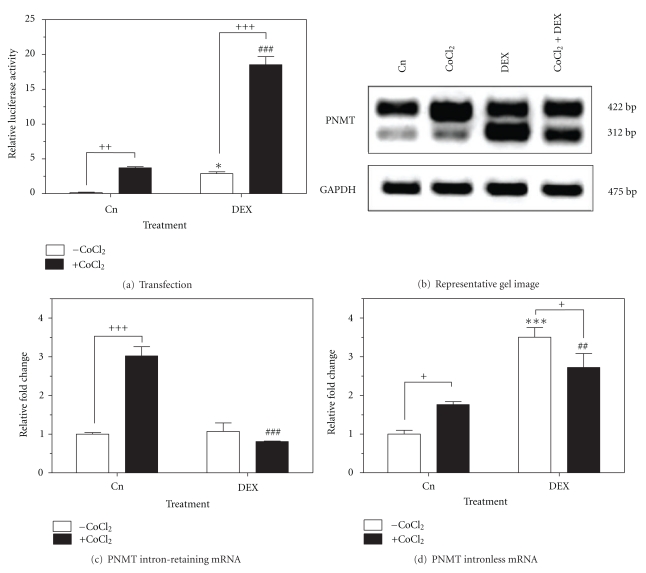
Effect of ROS on the hormonal regulation of the PNMT gene. PC12 cells were treated with 200 *μ*M CoCl_2_, 1 *μ*M dexamethasone or the combination of CoCl_2_ and dexamethasone for 24 h at 37°C. (a) Luciferase activity from cells transfected with the wild-type pGL3RP893 PNMT promoter luciferase reporter gene construct. (b) Representative image (*n* = 3) of PNMT (intron-retaining and intronless splice variants) mRNA levels from semiquantitative RT-PCR. GAPDH mRNA was used as a housekeeping control. (c) PNMT intron-retaining mRNA and (d) intronless mRNA. Data is normalized to GAPDH control. Data presented as relative fold change ± SEM from a minimum of three independent experiments. Significance defined as ^∗/+^ *P* < 0.05; ^++/##^ *P* < 0.01; ^∗∗∗/+++/###^ *P* < 0.001.

To determine whether the intracellular ROS generated by CoCl_2_ could potentiate the glucocorticoid activation of the PNMT gene, transient transfection assays and RT-PCR were performed on cells treated with 200 *μ*M of CoCl_2_, 1 *μ*M of dexamethasone and the combination of both drugs for 24 h. Transient transfection assays with a PNMT promoter-luciferase reporter gene construct harbouring the proximal −893 bp (pGL3RP893), revealed activation of the PNMT promoter following dexamethasone treatment (2.9-fold; *P* < 0.05), CoCl_2_  treatment (3.7-fold, *P* < 0.01), and the combination treatment (18.5-fold; *P* < 0.001) (Figure 3(a)). RT-PCR demonstrated increased expression of the intron-retaining splice variant of PNMT when treated with CoCl_2_ (3.0-fold; *P* < 0.001) and decreased expression when treated in combination with dexamethasone (0.8-fold; *P* < 0.001) (Figure 3(c)). Further, RT-PCR showed increased expression of the intronless splice variant of PNMT when treated with CoCl_2_ (2.7-fold; *P* < 0.05) compared to control and decreased expression when treated in combination with dexamethasone (2.7-fold; *P* < 0.01), compared to treatment with dexamethasone alone (3.5-fold; *P* < 0.001) (Figure 3(d)).

### 3.4. Effect of ROS on the Neural Regulation of the PNMT Gene

**Figure 4:**
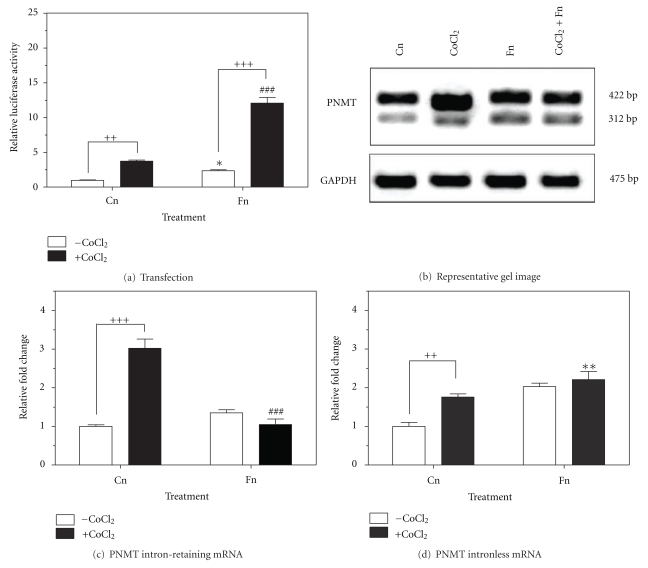
Effect of ROS on the neural regulation of the PNMT gene. PC12 cells were treated with 200 *μ*M CoCl_2_, 10 *μ*M forskolin or the combination of CoCl_2_ and forskolin for 24 h at 37°C. (a) Luciferase activity from cells transfected with the wild-type pGL3RP893 PNMT promoter luciferase reporter gene construct. (b) Representative image (*n* = 3) of PNMT (intron-retaining and intronless splice variants) mRNA levels from semi-quantitative RT-PCR. GAPDH mRNA was used as a housekeeping control. (c) PNMT intron-retaining mRNA and (d) intronless mRNA. Data is normalized to GAPDH control. Data presented as relative fold change ± SEM from a minimum of three independent experiments. Significance defined as *P < 0.05; ^∗∗/++^ *P* < 0.01; ^+++/###^ *P* < 0.001.

To determine whether intracellular ROS generated via CoCl_2_  treatment could potentiate the cholinergic activation of the PNMT gene, transient transfection assays and RT-PCR were performed on cells treated with 200 *μ*M of CoCl_2_, 10 *μ*M of forskolin and the combination of both drugs for 24 h. Transient transfection assays with a PNMT promoter-luciferase reporter gene construct harbouring the proximal −893 bp (pGL3RP893), revealed activation of the PNMT promoter following forskolin treatment (2.4-fold; *P* < 0.05), CoCl_2_  treatment (3.7-fold, *P* < 0.01), and the combination treatment (12.1-fold; *P* < 0.001) (Figure 4(a)). Combination treatment of forskolin and CoCl_2_ showed a decrease in intron-retaining PNMT mRNA levels (1.0-fold, *P* < 0.001) compared to CoCl_2_ treatment alone (Figures 4(b) and 4(c)). RT-PCR for the PNMT intronless splice variant demonstrated increased expression when treated with forskolin (2.0-fold, *P* < 0.01), compared to control (Figures 4(b) and 4(d)), however, showed no further increase when treated in combination with CoCl_2_.

## 4. Discussion

Epinephrine is found in the adrenal medulla, adrenergic neurons, the heart, the spleen, and the liver. It is involved in a variety of regulatory systems such as psychomotor activity, sleep, memory and the stress response [30]. In adrenergic cells, norepinephrine is N-methylated by the enzyme phenylethanolamine N-methyltransferase (PNMT) using S-adenosyl methionine as a co-substrate and a methyl donor to form epinephrine [12]. Understanding epinephrine production via its synthesizing enzyme PNMT is essential to elucidating its role as a neuroendocrine regulator in disease states [2]. Activation and regulatory mechanisms of the PNMT gene have been studied extensively in various systems. Recent studies demonstrate that PNMT is a stress-responsive enzyme with stressor-specific responses [31, 32]. Hypoxic stress is one of the many stressors that can stimulate the PNMT gene [17]. Tai et al., 2009, have previously demonstrated that hypoxia and CoCl_2_ stimulate the hypoxia inducible factor (HIF) 1*α* in PC12 cells to activate the PNMT promoter and PNMT [13]. In adrenomedullary chromaffin cells, trans-synaptic regulation of PNMT is mediated via neurotransmitter release from the splanchnic nerve, which leads to the activation of the PNMT gene [9]. Additionally, the biosynthesis of epinephrine in the adrenal medulla is dependent on high concentrations of glucocorticoids [33]. Glucocorticoids control PNMT post-translationally by indirectly preventing the degradation of the PNMT enzyme and by regulating the expression of PNMT mRNA [2]. The objectives of this study were to examine and elucidate the roles of reactive oxygen species (ROS), generated by the hypoxia mimetic agent CoCl_2_ on the hormonal and neural regulation of the PNMT gene. 

ROS exert critical intracellular functions, including signal transduction, gene transcription, and the regulation of guanylate cyclase activity in cells [34]. Intracellular levels of ROS have been demonstrated to augment during stress, which includes hypoxia [35]. The ROS nitric oxide (NO) is recognized as an essential intracellular messenger in the central and peripheral nervous systems. Previous studies have shown that NO leads to long-term upregulation of PNMT and other catecholamine biosynthetic enzymes, and that this is mediated by the cyclic GMP-dependent signaling pathway [36]. As such, we examined intracellular levels of ROS and extracellular levels of NO in PC12 cells exposed to hypoxia or treated with CoCl_2_. Our findings confirm that hypoxia and CoCl_2_ both increase intracellular levels of ROS and show that both treatments result in decreased extracellular NO. This demonstrates that CoCl_2_ acts as a hypoxia mimetic agent by increasing ROS in addition to the stabilization of HIF1*α* and is suggestive that NO is not involved in the induction of PNMT by CoCl_2_. 

Treatment of cells having elevated levels of ROS with known antioxidants, including *N*-acetylcysteine (NAC) and polyphenols, has demonstrated the ability to prevent ROS-induced responses [22]. Our findings confirm the upregulation of both the intronless and intron-retaining transcripts of the PNMT gene via ROS [31]. Furthermore, decreased intracellular levels of ROS and subsequent inhibition of PNMT transcription were observed when cells were treated jointly with CoCl_2_ and NAC. Since previous studies have demonstrated that CoCl_2_ mimics hypoxia by causing the stabilization of HIF proteins, our findings further confirm that NAC can effectively abolish the HIF1*α* stabilization by CoCl_2_, subsequently inhibiting the transcription of PNMT [37, 38]. In an effort to further elucidate the role of CoCl_2_-generated ROS on PNMT activation and transcription, we examined the combined effects of CoCl_2_ treatment with dexamethasone or forskolin, known hormonal and neural activators of the PNMT promoter. Similar to previous studies, we have demonstrated that both dexamethasone and forskolin increase the expression of the intronless splice variant of the PNMT gene [6, 9]. 

Glucocorticoids are steroid hormones released from the adrenal cortex and are the principle effectors in the stress response [39]. Glucocorticoid sensitivity has been reported for PNMT promoter activity in rat and bovine, and at least one putative glucocorticoid response element (GRE) has been identified for every species-specific PNMT gene [40]. The current study shows that PC12 cells transfected with the full-length promoter and treated with both CoCl_2_ and dexamethasone results in a synergistic effect and further drives transcription of the PNMT gene greater than the treatment with either drug alone. This response may be attributed to a previously reported redox mechanism in which CoCl_2_ increases stabilized glucocorticoid receptor (GR) protein within the cell [41]. Additionally, individual studies have identified an active −282 bp hypoxia response element (HRE) and −533, −759 and −773 bp GREs on the PNMT promoter [6, 13]. We hypothesize, that it is the combined stimulation of these elements that are giving rise to the observed synergistic activation of the PNMT promoter. Moreover, increased protein levels of the transcription factors, early growth response factor 1 (Egr-1) and specificity protein 1 (Sp1) by HIF1*α*, may further drive the PNMT promoter, giving rise to increased levels of cytoplasmic PNMT protein [13, 19]. Although, synergistic effect at the transcriptional level is not observed, a shift from the intron-retaining variant of PNMT to the intronless variant is present, suggesting further post-transcriptional regulation by CoCl_2_ and dexamethasone. 

Previous studies have demonstrated that forskolin treatment may lead to neural activation of the rat PNMT promoter via the protein kinase A (PKA) and protein kinase C (PKC) pathways, subsequently leading to increased nuclear protein levels of Egr-1 and Sp1 and increased cytoplasmic PNMT protein [7, 42, 43]. Our findings reveal that PC12 cells transfected with the full-length promoter and treated with both CoCl_2_ and forskolin results in a synergistic effect and further drives transcription of the PNMT gene greater than treatment with either drug alone. We attribute this response to the combination of multiple factors, mainly stabilized GR protein by CoCl_2_, increased activation of the HRE by HIF1*α*, and increased Egr-1 and Sp1 activation of the PNMT promoter. While we can currently only hypothesize on the exact mechanisms responsible for the synergistic activation of the PNMT promoter, future overexpression and knockdown experiments on key regulatory factors will permit for a better understanding of PNMT regulation. Similar to combination treatments with dexamethasone, we show a shift from the intron-retaining variant of PNMT to the intronless variant when treated with CoCl_2_ and forskolin. These findings suggest post-transcriptional regulation of PNMT by these two drugs. 

In summary, previous studies have elucidated and identified factors involved in the activation of the PNMT promoter via the hormonal and neural pathways. Additionally, the activation and synthesis of PNMT protein has been shown through the upregulation of HIF1*α* by the hypoxia mimetic agent CoCl_2_. For the first time, our findings show that hypoxia has synergistic effects on the activation of the PNMT promoter when combined with activation by dexamethasone or forskolin. Furthermore, this study shows that NO is likely not involved in the ROS-mediated transcription of the PNMT gene and that antioxidant treatment in combination with CoCl_2_ can abolish the ROS-mediated upregulation of the PNMT gene.

## Figures and Tables

**Table 1:** The following primer sets were used for PNMT and glyceraldehyde-3-phosphate dehydrogenase (GAPDH).

Gene	Species	Accession number	Primer sequence (5′–3′)	Amplicon	No. of cycles	Annealing temp. (°C)
PNMT	*R. norvegicus*	X75333	CAGACTTCTTGGAGGTCAACCG	422 bp, 312 bp	35	58
			(forward)			
			AGCAGCGTCGTGATATGATAC			
			(reverse)			

GAPDH	*R. norvegicus*	BC059110	ATGGTGGTGCTGAGTATGTCG	475 bp	21	58
			(forward)			
			CATGTCAGATCCACAACGGATAC			
			(reverse)			
